# Modelling the microelimination of chronic hepatitis C in the canton of Bern, Switzerland: Reaching the Swiss Hepatitis Strategy goals despite the impact of the COVID 19 pandemic

**DOI:** 10.1371/journal.pone.0272518

**Published:** 2022-08-12

**Authors:** Olivier Schorr, Sarah Blach, Christine Thurnheer, Christian Ruis, Jean-Francois Dufour

**Affiliations:** 1 Master of Public Health, University Basel, University Bern & University Zurich, Zurich, Switzerland; 2 Medical Affairs Department, Gilead Sciences, Zurich, Switzerland; 3 CDA Foundation, Lafayette, Colorado, United States of America; 4 Department of Infectious Diseases, University Hospital Bern, University of Bern, Bern, Switzerland; 5 Department of Gastroenterology, Spital STS AG, Thun, Switzerland; 6 Private Practice, Pully, Switzerland; Medizinische Fakultat der RWTH Aachen, GERMANY

## Abstract

**Aims of the study:**

Since 2014, the Swiss Hepatitis Strategy (SHS) has targeted the elimination of Hepatitis C Virus (HCV) in Switzerland. The epidemiology of HCV is diverse across Swiss cantons, therefore cantonal-level screening and treatment strategies should be developed. This study aimed to identify scenarios to achieve HCV elimination in the canton of Bern by 2030.

**Methods:**

A preexisting Markov disease burden model was populated with data for Bern, and used to forecast the current and future prevalence of HCV, annual liver-related deaths (LRDs), and incidence of hepatocellular carcinoma and decompensated cirrhosis until 2030. Scenarios were developed to assess the current standard of care and potential long-term impact of the COVID-19 crisis on the HCV infected population. Additionally, potential scenarios for achieving the WHO 2030 targets and the SHS 2025 and 2030 targets (reduction of new cases of HCV, HCV-related mortality and viremic HCV cases) were identified.

**Results:**

In 2019, there were an estimated 4,600 (95% UI: 3,330–4,940) viremic infections in the canton of Bern and 57% (n = 2,600) of viremic cases were diagnosed. This modelling forecasted a 10% increase in LRDs (28 in 2020 to 31 in 2030) with the current standard of care and a 50% increase in LRDs in a scenario assuming long-term delays. To achieve the WHO and SHS targets, the canton of Bern needs to increase the annual number of patients diagnosed (from 90 in 2019 to 250 per year in 2022–2024 [WHO], or 500 per year in 2022–2025 [SHS]) and treated (from 130 in 2019 to 340 per year in 2022–2024 [WHO] or 670 per year in 2022–2025 [SHS]).

**Conclusions:**

The SHS goals and the WHO targets for HCV elimination can be achieved in the Swiss canton of Bern by 2030; however, not at the current pace of screening, linkage to care and treatment.

## Introduction

The World Health Organization (WHO) refers to Hepatitis C virus (HCV) infection as a “viral time bomb” [1]. This is not only due to the asymptomatic clinical course, even in advanced stages of liver disease, but also to the frequency with which it is overlooked. Out of 100 people infected with HCV, over 75–85 develop chronic HCV infection if left untreated [2]. Of these, 10% to 20% of patients progress to cirrhosis over a period of 20 to 30 years, with subsequent annual risk of 1–5% for developing hepatocellular carcinoma (HCC) and 3–6% for hepatic decompensation [3]. Chronic HCV infection can additionally lead to cryoglobulinemia, which may cause vasculitis, glomerulonephritis and B-cell non-Hodgkin’s lymphoma. Insulin resistance, diabetes mellitus and arteriosclerosis also occur at higher rates in chronic hepatitis patients [4].

Viral hepatitis is recognized by the WHO as a global epidemic, with nine times more people infected with HCV than with HIV [5]. Additionally, deaths from hepatitis have been increasing over the past two decades [5]. To address this, the WHO set a goal to eliminate viral hepatitis as a major public health threat by 2030 [6]. In 2014, a similar effort was undertaken by the “Swiss Hepatitis” network to devise the Swiss Hepatitis Strategy (SHS) [7]. This strategy targets the elimination of HCV in Switzerland with a goal of reducing the socio-economic impact of viral hepatitis, decreasing transmission, and reducing associated morbidity and mortality.

A previous study showed that HCV incidence and prevalence were decreasing in Switzerland [8]; but without a significant future expansion in treatment, morbidity and mortality would increase. The highest incidence of HCV was observed during the late 1980s and 1990s, and since complications of end-stage liver disease usually occur 20–30 years after infection, at present, there is a significant HCV-related burden on healthcare and the economic system [8]. The European Association for the Study of the Liver (EASL) recommends that treatment-naïve and treatment-experienced patients with recently acquired or chronic HCV infection be offered treatment without delay. The goal of therapy is to cure chronic HCV infection to prevent the complications described above [9]. Another article, published in collaboration with the Swiss Federal Office of Public Health (FOPH) [10], evaluated the clinical and economic burden of HCV intervention strategies in Switzerland over the next 15 years. The authors concluded that expanding treatment eligibility to all patients regardless of fibrosis stage, combined with increasing the number of treated patients may have a positive impact on disease burden and may be cost-effective [10].

Previous studies have shown that the epidemiology of HCV is diverse across Swiss Cantons [11, 12]. Therefore, targeted and evidence-based screening as well as locally defined treatment strategies should be developed at a cantonal level. The primary objective of this present study was to describe the epidemiology of HCV in the canton of Bern as well as current screening, linkage to care and treatment standards. We additionally aimed to present two scenarios reflecting a status quo in screening and treatment rate by 2030, with or without the negative impact of the Coronavirus disease 2019 (COVID-19) crisis on treatment initiation. In two further scenarios, we modelled and identified the increases in diagnosis, linkage to care and treatment rates required in the canton of Bern to reach the WHO and the Swiss Hepatitis Strategy elimination targets by 2030.

## Materials and methods

First, historical HCV epidemiological data were collected for the canton of Bern and entered in a preexisting Markov model [13–15]. The model inputs and outputs were reviewed and revised in consultation with local experts (the term expert will be exclusively used in this manuscript to describe the Delphi expert group) during two expert meetings. Four scenarios were developed to evaluate the HCV-related morbidity and mortality. The outputs of the four scenarios were then compared.

The focus of this analysis was on the canton of Bern, situated in central Switzerland. The population of the canton was 1’039’474 at the end of 2019, representing approx. 12% of the Swiss population [16]. There are two cities in the canton with >50’000 inhabitants, corresponding to 18.4% of the canton’s population. This percentage of urban population is comparable to that of the entire Swiss population.

### Hepatitis C disease burden model

A previously described Markov state transition model populated with data for Switzerland [17] was updated to reflect the geographical scope and epidemiological situation of the canton Bern. In addition to the brief description provided here, a comprehensive description of the HCV Bright model has been published previously [13–15] and can be found in S1 Appendix. The model was constructed in Microsoft Excel® (Microsoft Corp., Redmond, WA) to quantify the size of the HCV-infected population from 1950–2030 by the liver disease stages. The size and impact of the HCV infected population prior to 1950 were considered negligible for the purposes of the analysis [14]. The Excel® optimization add-in, Frontline Systems’ Solver, was used to calculate the number, age and gender distribution of the annual acute infections (the add-in solves the equation where New Infections is the unknown variable; this can be found in S1 Appendix as equation 2). This disease progression model was used to estimate the annual numbers of acute infection that progress to chronic infection, to worse fibrosis stages, cirrhosis, hepatocellular carcinoma and eventually death.

The progression of these HCV cases, stratified by age and sex, was followed and adjusted for mortality and cure. In this analysis, the model was populated considering previously published data from other regions in Switzerland and global historical parameters [8, 11]. Adjustments were made based on feedback from experts and the model was calibrated with assumptions specific for the canton of Bern (as described below).

### Sensitivity analysis

The model was set up for sensitivity and Monte Carlo analysis using Crystal Ball® (Oracle, Austin, Texas, USA) [14]. We use Beta-PERT distributions for all uncertainty inputs (β-PERT allowed for non-symmetrical distributions based on the collected data) [14]. The progression rate from acute HCV infection to spontaneous clearance as well as the other progression rates and excess mortality rates (for previously transfused patients and injection drug users) are not country specific, but global inputs in the model [14].

### Data collection for definition of parameters

Data collection for the Bern model was based on three complementary strategies, which are described in more detail in S2 Appendix: (i) review of online resources (Federal Office of Public Health [FOPH], WHO, Swiss Hepatitis C cohort Study) to capture non-indexed sources of relevant data; (ii) PubMed literature database search from 2013 forward to identify recent publications with keywords HCV, prevalence and Switzerland (and if available: Bern) (A detailed description of the search criteria for the literature search can be found in S2 Appendix); (iii) gathering of empirical data on HCV-associated new HCC cases, liver transplants, percentages of HCC and liver transplantations related to HCV, annual number of newly diagnosed patients and annual number of treatments in Bern. The accuracy of the input data was discussed during a Delphi process. For the Delphi meeting, cantonal centers of high interest for this project were consulted to gain a better understanding of the situation in the canton of Bern. Two Delphi meetings were organized (September 2, 2020 and November 11, 2020) with 3 and 2 cantonal experts, respectively. Actively participating HCV experts included one hepatologist from Inselspital Bern and a gastroenterologist from Hospital Thun (the former having a rather urban and the latter a rather rural catchment area), and, in the first meeting, an infectiologist associated with both Inselspital Bern and the heroin-assisted treatment center KODA in Bern. The plausibility of model outputs was additionally validated by the experts. Finally, the obtained data and outputs were analyzed reviewed by an external specialist group from the Swiss Hepatitis association.

### Definition of parameters applicable to the canton of Bern

#### Prevalence

The exact data on the HCV prevalence for the canton of Bern were not available in the FOPH database or the cantonal database [18]. Therefore, they were estimated from the available national prevalence data from 2015 [19]. An expert consensus deemed the canton of Bern as representative of the Swiss population. Hence, the viremic prevalence (presence of anti-HCV antibodies in blood) in the canton of Bern was estimated at 0.49% (95% confidence interval [CI] 0.45–0.54%) [19]. The prevalence data for this study were compared and double-checked for consistency with published data from the cantons of Zurich, Geneva and Sankt Gallen [11] (Table 1).

**Table 1:** HCV prevalence in Switzerland and selected cantons for 2015.

Analysis	Viremic prevalence (2016) (95% CI)	Viremic cases in 2016 (95% CI)
**Switzerland**	0.49% (0.45% - 0.54%)	39,500 (36,000–43,000)
**Bern**	0.49% (0.45% - 0.54%)	5,070 (4,660–5,590)
**Geneva**	0.65% (0.59% - 0.71%)	3,260 (2,960–3,560)
**St. Galen**	0.49% (0.45% - 0.54%)	2,790 (2,600–3,130)
**Zurich**	0.73% (0.68% - 0.81%)	10,800 (9,900–11,900)

**Fig 1:**
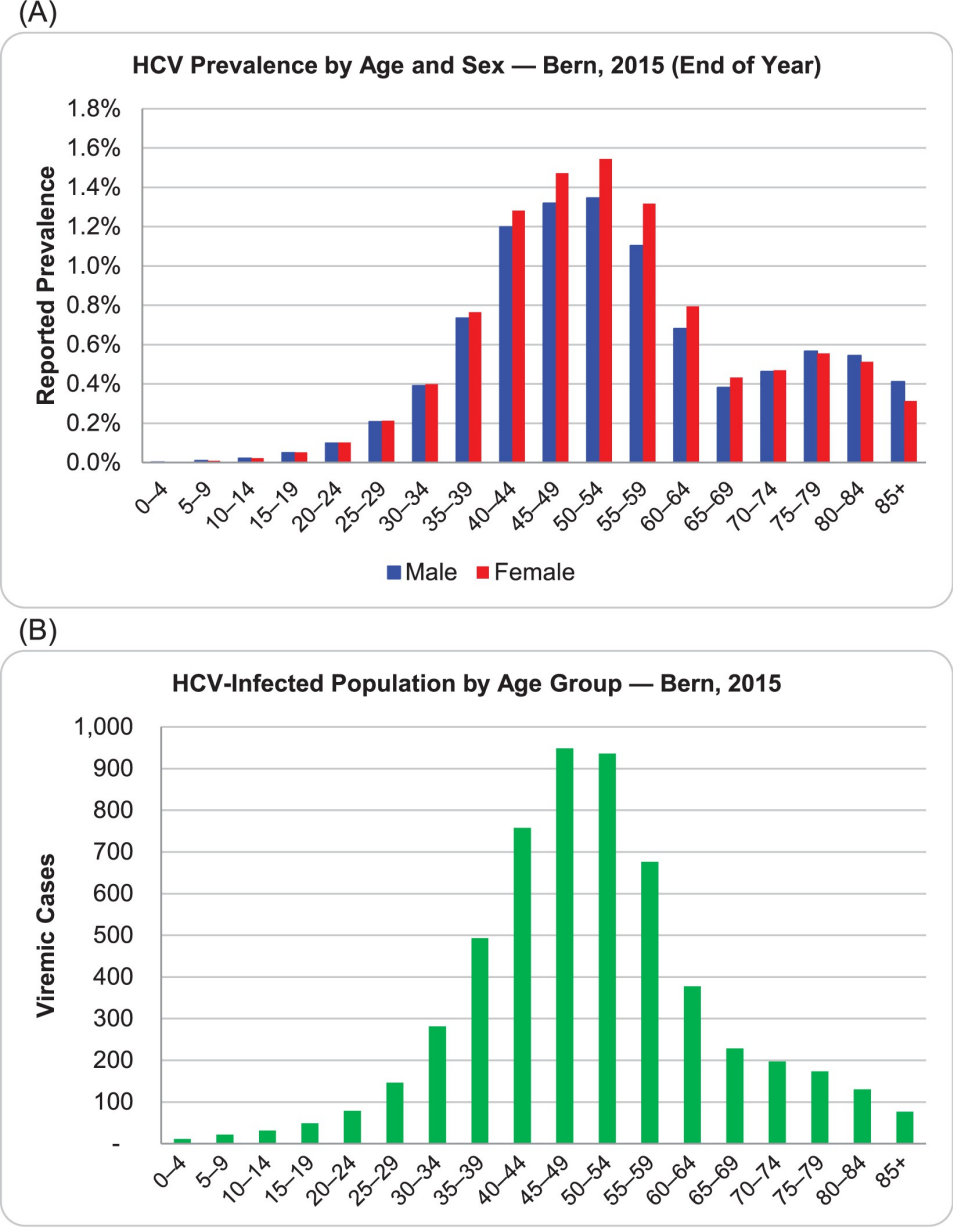
HCV prevalence by age and sex (A) and HCV infected population by age group (B) (canton of Bern). Adapted from Federal Office of Public Health, numbers and statitics [18].

The total number of HCV infections was calculated using the prevalence multiplied by the population of the canton of Bern [16]. To estimate by age and sex we could not find published data for the canton of Bern, so we used the prevalence by age and sex for Switzerland [18]. The resulting distribution was weighted to the 2015 prevalence estimate (Fig 1).

#### Genotype

HCV genotype data were calculated with the number of all diagnosed patients from the Inselspital Bern up to 2020 (Prof. J.-F. Dufour, personal communication). These cantonal data are comparable to the national data from the Swiss Hepatitis C cohort Study (Table 2) [20].

**Table 2:** HCV genotype distribution in Switzerland [20] and in Bern.

Genotype	1a	1b	1	2	3	4	5	6	Unknown
**Switzerland**	15.2%	13.7%	15.2%	7.2%	23.6%	9.0%	0.1%	0.1%	15.7%
**Bern**			49.5%	8.0%	28.8%	12.7%	0.3%	0.7%	

#### Estimation of diagnosed patients (newly and historical)

There were 4,300 total historical HCV notifications (acute and non-acute cases) reported from the canton of Bern to the FOPH from 1988 to 2015 [18]. However not all people who have been previously notified by FOPH are still infected with HCV. To calculate the number of people who were diagnosed and are still HCV positive, we adjusted for viremia, mortality and cure. Additionally, there were 116 newly cases reported for the canton of Bern in 2019 [18]. Assuming a viremic rate of 79.7% [21] there were 90 viremic newly diagnosed cases in 2019.

#### Incidence

**Fig 2:**
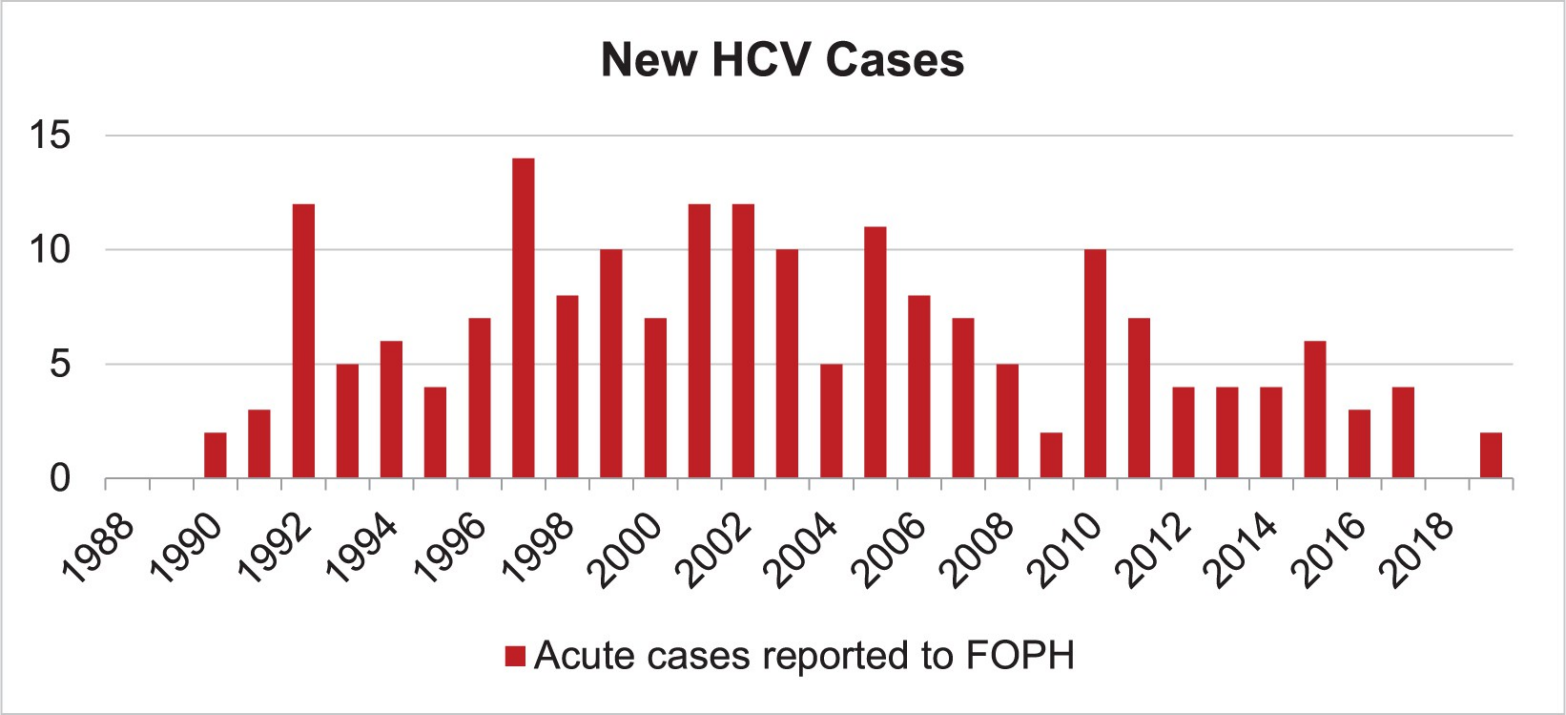
Acute new cases reported to FOPH for the canton of Bern. Adapted from Federal Office of Public Health, numbers and statitics [22].

The true number of new HCV infections is difficult to estimate. Acute HCV cases are notified to the Swiss FOPH, and in 2019, only 2 acute HCV cases were confirmed according to the Bern database [22]. Since acute cases are verified by cantonal medical officers, these are considered to be true new infections [22] (Fig 2). The FOPH differentiates between acute infections (incidence) and newly diagnosed cases, which contains some cases who may have been newly infected some years ago. However, the acute notified cases are likely to underestimate true incident infections, because most patients will not be diagnosed with HCV for many years after they are exposed. Additionally, HCV-infected people may enter the country through migration.

In order to estimate the total number of new infections each year in Bern, we first identified the shape of the trend in new infections over time. In Switzerland, new infections were expected to peak before blood screening was available in the early 1990s [19], which is assumed to be similar for Bern. In more recent years, the trend in acute infections reported to the FOPH was used to determine whether new infections were generally increasing or decreasing.

Once the relative trend in new infections over time was defined, the scale was calculated to match the total number of prevalent infections. This returned a value of around 55 new infections in 2020 in the canton. Based on prior modeling for Switzerland to estimate the number migration-related new infections [8, 11], we conclude that around 50 of the 55 annual new infections in Bern would be due to migration, while the other 5 infections would be true incident infections occurring within the canton in 2020.

#### Historically treated patients

The number of patients initiated on Direct Antiviral Agent (DAA) treatment annually was accessed through a variety of complementary sources, including the Swiss Pharmacist Cooperative (OFAC), the Swiss National Pharmacy Service (MediService), IQVIA (private company monitoring and forecasting sales) and industry data sources. In the OFAC data, the number of DAA units distributed within the pharmacy channel as well as anonymized patient starts can be determined on a monthly basis. An extrapolation using IQVIA sales data for the canton of Bern concludes the total number of patients initiated on a combination of DAAs. These combined data specific for Bern were used to calculate the number of patients treated by year in the canton (see S1 Appendix). An expert consensus during the Delphi process was used to check for consistency of these numbers (Table 3).

**Table 3:** Estimation of the number of patients annually treated with Direct Antiviral Agent (DAA) in the canton of Bern.

Year	2015	2016	2017	2018	2019
**DAA-treated patients per year (canton of Bern)**	270	293	245	190	126

#### Liver transplants

**Fig 3:**
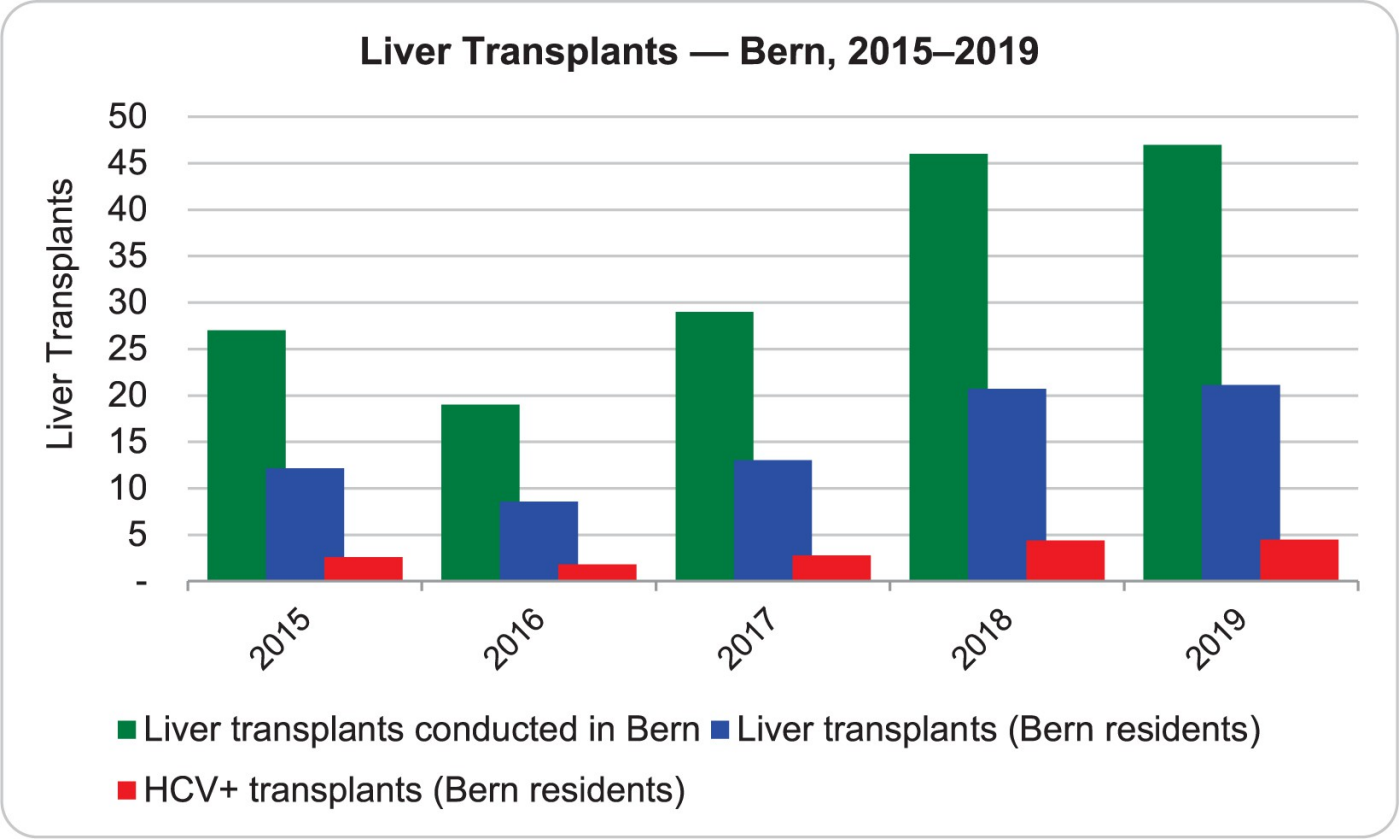
Liver transplants in Bern (2015–2019). Adapted from Swisstransplant preliminary statistics 2019 [23].

The number of liver transplants conducted in Bern from 2015–2019 was available through Swisstransplant [23]. The Liver Transplant Center Bern is one of three centers in Switzerland authorized by the FOPH to perform organ transplantations and therefore does not only transplant patients from Bern but also patients from surrounding cantons, but data on the patients’ cantons of origin are not published. During the consensus meetings, the expert panel estimated that approximately 45% of transplants in the Bern center are conducted for cantonal residents (Prof. J.-F. Dufour, personal communication). Moreover, the proportion of approximately 21% of liver transplants attributable to HCV was estimated from data from the transplant cohort and from the Geneva University Hospital data published previously [10], and confirmed during the expert discussion for this analysis (Fig 3).

### Scenarios

Four scenarios were developed to evaluate HCV disease burden in the canton of Bern: the first two scenarios studied the impact of no changes in the standard of care or the impact of a long term COVID-19 crisis. The last two scenarios were modelled with increased diagnosis, linkage to care and treatment rates to achieve the WHO and the SHS targets.

The first scenario “2020 Base” presumes that the current standard of care in the canton would continue up to 2030. In this scenario, the COVID-19-related impact on treatment rates would only affect the year 2020. After 2020, the number of patients initiated on treatment was assumed to rebound and remain constant at the 2019 level (before the COVID-19 crisis). In this scenario, we also assumed that the number of patients diagnosed annually would slightly decrease with no improvement in linkage to care, screening strategies and case finding. All chronic HCV patients have access to treatment regardless of fibrosis stage, the treatment access expansion from fibrosis stage ≥F2 to ≥F0 has been in effect since the end of 2017. The treatment rate is modeled to remain constant in this specific scenario. From 2020, the response to treatment (Sustained Virological Response, SVR) was set to an average of 98%, regardless of genotype or fibrosis stage. This cure rate is typical for patients receiving the latest DAAs. Under this scenario, 130 patients would be treated each year (except in 2020, the year impacted by COVID-19, when only 40 patients were assumed to have been treated). Finally, although new infections due to immigration are considered in the model, the new infections in this table only reflect the truly new local infections (excluding the new infections due to immigration) for the purposes of evaluating progress toward the WHO elimination targets (Table 4).

**Table 4:** Inputs by scenario for the canton of Bern 2015–2030.

**2020 Base**	**2015**	**2019**	**2020**	**2021**	**2022**	**2025**
**Treated**	110	130	40	130	130	130
**Newly Diagnosed**	270	90	90	90	90	80
**Fibrosis Stage**	≥F2	≥F0	≥F0	≥F0	≥F0	≥F0
**New Infections**	21	9	5	2	1	1
**Treated Age**	15–85+	15–85+	15–85+	15–85+	15–85+	15–85+
**SVR**	95%	95%	98%	98%	98%	98%
**COVID long term delays**	**2015**	**2019**	**2020**	**2021**	**2022**	**2025**
**Treated**	110	130	40	40	40	40
**Newly Diagnosed**	270	90	90	90	90	80
**Fibrosis Stage**	≥F2	≥F0	≥F0	≥F0	≥F0	≥F0
**New Infections**	21	9	5	2	2	1
**Treated Age**	15–85+	15–85+	15–85+	15–85+	15–85+	15–85+
**SVR**	95%	95%	98%	98%	98%	98%
** WHO Targets**	**2015**	**2019**	**2020**	**2021**	**2022**	**2025**
**Treated**	110	130	40	170	340	300
**Newly Diagnosed**	270	90	90	200	250	90
**Fibrosis Stage**	≥F2	≥F0	≥F0	≥F0	≥F0	≥F0
**New Infections**	21	9	5	2	1	1
**Treated Age**	15–85+	15–85+	15–85+	15–85+	15–85+	15–85+
**SVR**	95%	95%	98%	98%	98%	98%
**SHS 2025+2030**	**2015**	**2019**	**2020**	**2021**	**2022**	**2026**
**Treated**	110	130	40	250	670	400
**Newly Diagnosed**	270	90	90	230	500	40
**Fibrosis Stage**	≥F2	≥F0	≥F0	≥F0	≥F0	≥F0
**New Infections**	21	9	5	2	1	1
**Treated Age**	15–85+	15–85+	15–85+	15–85+	15–85+	15–85+
**SVR**	95%	95%	98%	98%	98%	98%

The second scenario “long-term COVID-19 delays” assumed that the number of patients treated in 2020 dropped by 70% from 2019 (same level as in in the base case scenario) and that the number of patients initiated on treatment would never recover and remain at 40 patients treated per year. All the other inputs were the same as in the 2020 base (Table 4).

The third scenario analyzed was the “WHO targets” scenario. This scenario was based on the first global health sector strategy on viral hepatitis developed by WHO; which was modelled to reach a strategy that would contribute to the achievement of the 2030 Agenda for Sustainable Development. The WHO Global Health Sector Strategy on Viral Hepatitis 2016–2021 set targets for the elimination of HCV as public health threats by 2030, with a 90% reduction in new chronic HCV infection, and a 65% reduction in viral hepatitis C deaths by 2030 [6] (Table 4).

The fourth scenario analyzed was the “Swiss Hepatitis Strategy (SHS) 2025 and 2030”. Considering the situation in Switzerland, a network of 80 specialists launched the Swiss Hepatitis Strategy, under the umbrella of the association “Swiss Hepatitis” (www.hepatitis-schweiz.ch). The strategy includes additional targets that would help reduce the disease burden in Switzerland (e.g., cases of liver cancer and transplants). The three main targets are: (i) reduction of new cases of HCV by 60% by 2025 and 95% by 2030; (ii) reduction of HCV-related mortality by 50% by 2025 and 95% by 2030; and (iii) reduction of viremic HCV cases by 60% by 2025 and 95% by 2030 [7] (Table 4).

## Results

**Fig 4:**
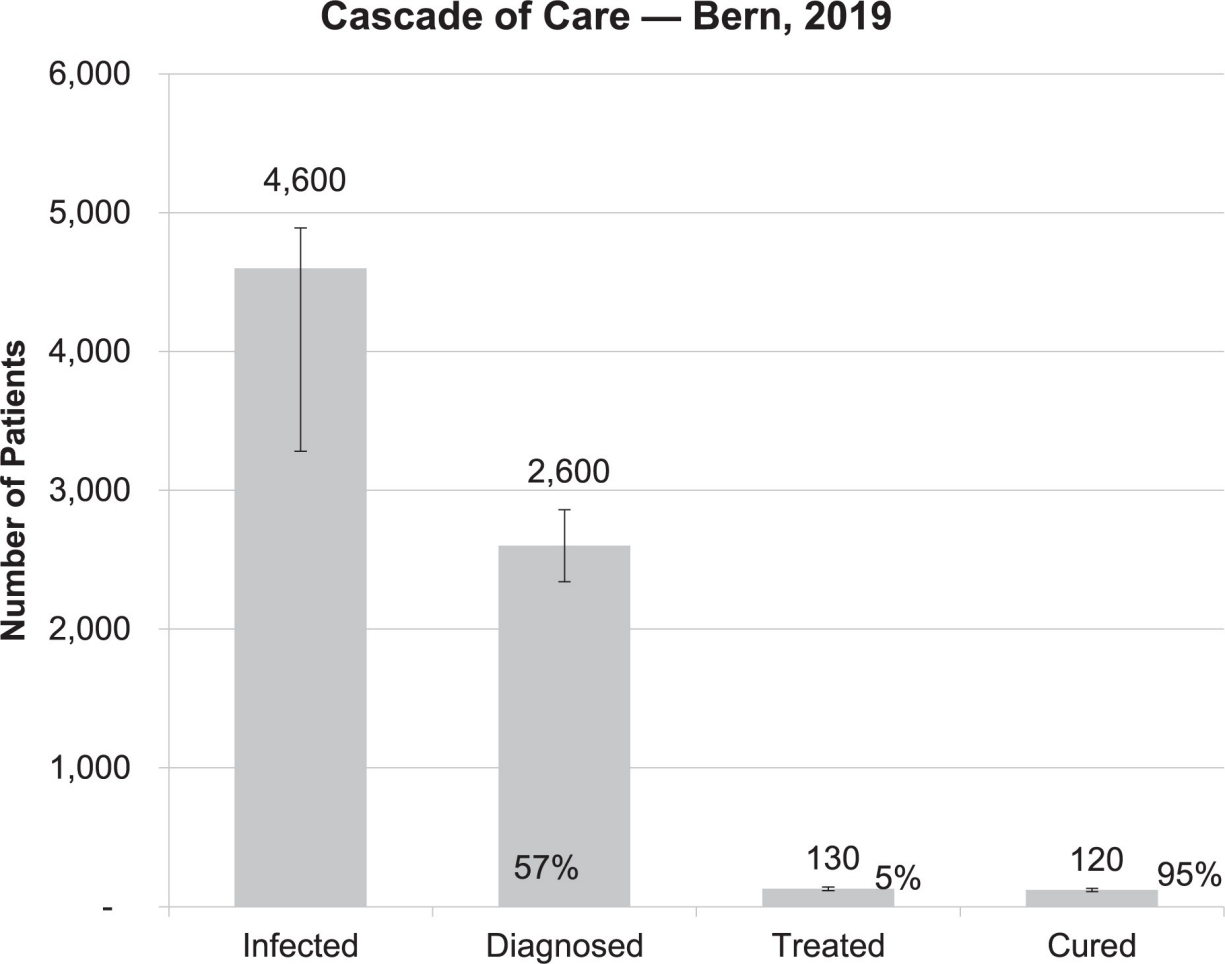
Cascade of care, canton of Bern (2019) including total viremic infections, diagnosed, treated and cured (the bars represent the 95%UI).

In 2015, the estimated viremic prevalence in the canton of Bern was 0.49% (same as the Swiss prevalence) with 5’070 viremic cases (95% UI: 4’660–5’590) [19]. Using this input, the model predicted that there were still 4’600 viremic cases in the canton in 2019 (Fig 4). The 95% uncertainty interval (UI) ranged from 3’330–4’940; this uncertainty range was calculated using a Monte Carlo simulation of 1’000 trials, with the inputs described previously.

**Fig 5:**
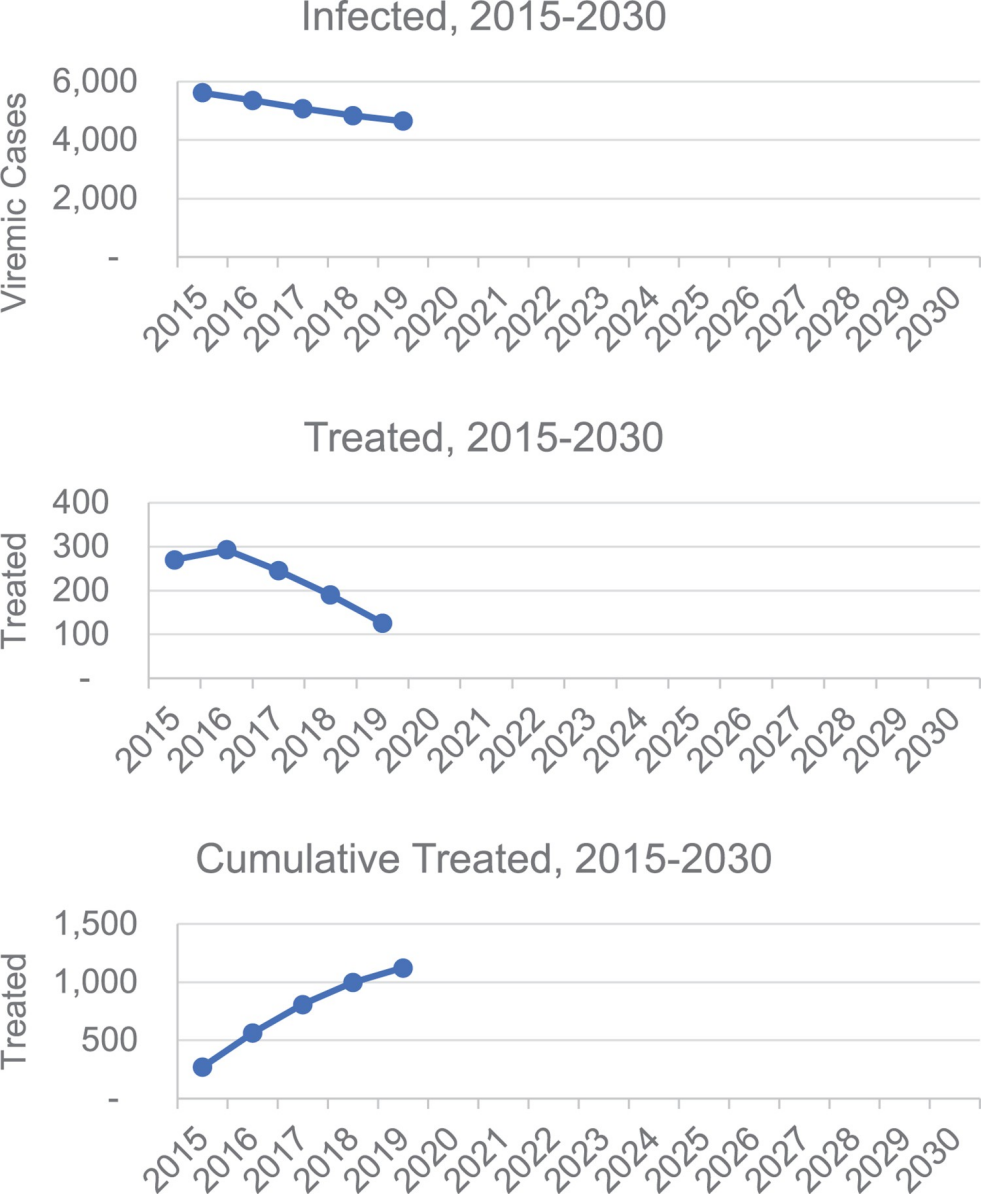
Number of viremic infected patients (prevalence), annual treated and cumulated treated between 2015 and 2030 in the canton of Bern.

The HCV cascade of care for the canton of Bern is presented here (Fig 4). In Bern, 57% of the 4’600 infected viremic patients were diagnosed (model output, validated by Delphi process) (see S1 and S2 Appendices). Only 5% (n = 130) of these 2’600 diagnosed patients received treatment in 2019. With an estimated 95% cure rate, approximatively 120 patients were cured. The number of infected patients in the canton decreased between 2015 and 2019 (Fig 5). Finally, as presented in Fig 5, the number of treated patients decreased starting in 2017 (after treating the higher fibrosis patients and patients already in care, the urgency to treat and availability of patients linked to care decreased) and the cumulated number of patients treated during the previous five years is more than 1’100.

**Fig 6:**
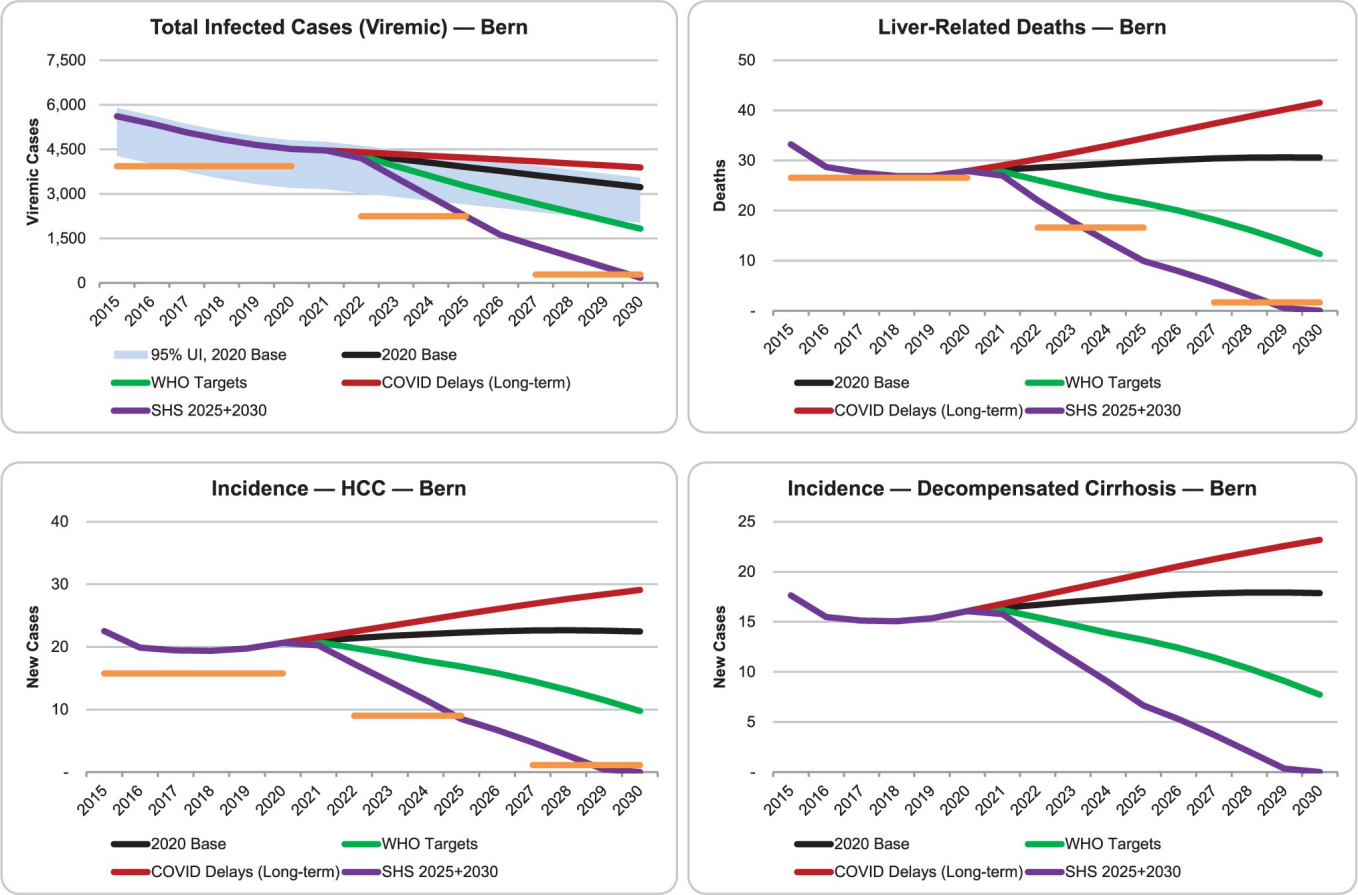
Total viremic infections, liver-related death, incidence of hepatocellular carcinoma (HCC) and incidence of decompensated cirrhosis by scenario for the canton of Bern. (orange bars: Swiss Hepatitis Strategy 2020, 2025 and 2030 targets; where applicable).

The total number of viremic infections, liver related death, incidence of HCC and incidence of decompensated cirrhosis by scenario for the canton of Bern are depicted in Fig 6.

### 2020 Base scenario

Under the 2020 Base scenario, where current standard of care would continue up to 2030, the model forecasted a reduction of total viremic cases in the canton of Bern from 4’520 in 2020 to 3’220 in 2030, representing a decrease of 28%. With a stable level of 130 treatments per year and a steady new diagnosis rate (approx. 90 patients/year), the number of liver related deaths would slightly increase in this scenario (28 in 2020 to 31 in 2030; +10%). This scenario forecast a minor impact on incidence of HCC (21 in 2020 to 22 cases in 2030; +5%), as well as a slight impact on the incidence of decompensated liver cirrhosis (16 in 2020 to 18 cases in 2030; +12%; Fig 6).

### Long-term COVID-19 delays scenario

This scenario assumeed that the number of patients treated in 2020 would drop by 70% compared to 2019 and never recover following the COVID-19 crisis. With 40 treatments per year in Bern for one decade, the model forecasted a 14% decrease in viremic cases (4’520 viremic cases in 2020 to 3’890 in 2030; i.e., 620 patients). Assuming the same screening rates as in the base case (approx. 80 to 90 new HCV diagnoses per year) the number of liver-related deaths would increase by 50% between 2020 and 2030 (28 to 45 cases). Finally, the model predicted that the incidence of HCC would increase in this period from 21 to 29 cases (+38%). Likewise, the number of decompensated cirrhosis cases would increase by 43% (16 cases in 2020 to 23 cases in 2030; Fig 6).

### WHO targets scenario

This scenario was characterized by two main targets: 90% reduction in new chronic HCV infection and a 65% reduction in HCV-related deaths by 2030 [6]. In order to achieve all of the WHO targets towards elimination of viral hepatitis, the input settings had to be increased: the number of chronic HCV infections required to be detected from 2021 was 220, reaching a peak of 250 new diagnoses in 2022–2024 (Table 4). Assuming a scale up of treatment to 200 in 2021, 340 in 2022–2024 and 300 therapies thereafter, the model estimated a 60% reduction of viremic cases from 2020 to 2030 (4’520 to 1’830 cases). With these diagnosis and treatment rates, the number of liver related deaths was forecast to decline by 66% between 2015 and 2030, thus reaching the 65% mortality reduction target. The mortality reduction between 2020 and 2030 was forecast to be 51% (a decrease from 28 to 11). In this scenario, the number of new infections would drop from 21 in 2015 to 1 in 2030. In this scenario, the incidence of both HCC and decompensated cirrhosis would drop by 50% between 2020 and 2030 (HCC: decrease from 21 to 10 cases; decompensated cirrhosis: decrease from 16 to 8). (Fig 6)

### Swiss Hepatitis Strategy (SHS) 2025 and 2030 scenario

In this scenario, there were three main targets to be reached: (i) reduction of new HCV cases by 60% in 2025 and 95% in 2030; (ii) HCV-related mortality reduction by 50% in 2025 and 95% in 2030; and (iii) reduction of the viremic HCV cases by 60% in 2025 and 95% in 2030 [7]. To reach these targets in this Markov modelling, the input settings had to be increased: the yearly number of patients to be treated in 2021 was set to 250, for 2022–2025 to 670 and to 400 thereafter (Table 4). The strategy would need to diagnose 230 new HCV patients in 2021 and to reach a peak of 500 new HCV cases during the following 4 years. The incidence of HCV would then drop radically (from 21 new cases in 2015 to 1 in 2030). The prevalence would decrease to less than 180 viremic infections remaining in 2030 in this forecast (95% reduction). The number of liver-related deaths, HCC incidence as well as cases of decompensated cirrhosis would each drop to nearly zero starting in 2029. The SHS targets depicted in Fig 6 (orange bars) would all be met; the target reached the earliest in the modelling of this scenario would be the one for liver-related death, with a 50% mortality decrease achieved by 2023: 14 liver-related deaths would be saved each year in 3 years’ time.

As shown in Table 5, COVID 19 could have a significant long-term impact on the number of treated patients (only 460 treated between 2020 and 2030). With the WHO and the SHS scenarios, the cumulative number of patients diagnosed could reach a total of 1’600 and 2’400 new diagnoses during the period 2020–2030 respectively, and a total of 3’000 and 4’700 patients, respectively, could receive treatment.

**Table 5:** Cumulative number of patients diagnosed and treated by scenario from 2020 to 2030.

2020–2030	Newly Diagnosed	Treated
**2020 Base**	920	1,300
**Long-term COVID-19 delays**	920	460
**WHO Targets**	1,600	3,000
**Swiss Hepatitis Strategy 2025 and 2030**	2,400	4,700

### Sensitivity and uncertainty analyses

In a sensitivity analysis, the progression rate from acute HCV infection to spontaneous clearance was responsible for the highest variance in 2019 viremic prevalence. The viremic prevalence in 2016 was the variable responsible for the second largest variance in the 2019 viremic prevalence. These numbers published by the FOPH are specific to Switzerland [19] and were scaled down for the canton of Bern. If the viremic prevalence was at the upper range the FOPH estimate (5,590 instead of 5,070), a total number of 4,900 rather than 4600 viremic infections in 2019 would be expected (For additional details, see S3 Appendix).

## Discussion

This modelling analysis investigated the epidemiology of hepatitis C in the canton of Bern in Switzerland, studied the potential long-term impact of the COVID-19 crisis on the HCV-infected population and identified potential scenarios for achieving the WHO and the SHS targets, respectively.

### Comparison with international research and policy implications

In 2016, Australia was one of the first countries in the world to grant access to the newest highly effective therapies against HCV without any restrictions, resulting in a rapid adoption of the new therapies: 32’600 people initiated DAA in 2016 [24]. Australia, with a population 25 million inhabitants, has three times more inhabitants than Switzerland and the canton of Bern represents 12% of Switzerland. A simple calculation shows that a treatment rate similar to the Australian strategy would correspond to 1’300 treatments per year for the canton. The study published in 2019 by Kwon *et a*l [24] suggests that Australia is on track to achieve WHO elimination targets secondary to this rapid increase in treatments. However, according to that analysis, Australia is in the position of having a high percentage of the HCV population diagnosed (approx. 81%). Our modelling suggests that 57% of patients are diagnosed in the canton of Bern.

The Australian example (with focus on testing, harm reduction programs, prevention and treatment) demonstrates that elimination is achievable. Hence, this target could also be reached in Switzerland. The Swiss harm reduction and prevention programs are world-renowned and rated as visionary; they should be continued [25]. The focus for Switzerland should therefore be on testing and linkage to care.

The Swiss diagnosis rate could be enhanced by efforts to lower the threshold for HCV counselling and testing in pharmacies and at the general practitioner (GP) level. In Switzerland, prescription of DAA medication is restricted to specialists (infectiologists, gastroenterologists/hepatologists as well as some addiction medicine specialists). The current maximum number of physicians who are authorized and willing to treat HCV patients in the canton of Bern is approximately 40 (assumptions derived from a market analysis). This number is too low to reach the targets. HepCare, a flagship project of the association Swiss Hepatitis, enables HCV therapy in GP offices [26]. These physicians often have trust-based relationships with their patients. The pragmatic approach of this program allows specialists to prescribe DAA based on the patient records and to support the GP and any psychiatrist involved during the treatment. A low threshold strategy was deemed very impactful as well as cost-effective in the Australian setting [27]. If the HepCare program is successfully implemented and scaled up in the canton of Bern, this could be a future game changer.

### COVID-19 long term impact

The impact of COVID-19 on the management of non-COVID-19 patients was described in this modelling with HCV as an example. In this scenario, we assumed the decrease in HCV treatments would be entirely due to consequences of the COVID-19 pandemic, instead of a possible decrease in the number of HCV positive patients awaiting treatment. If the HCV treatment rate does not recover following the pandemic, we estimated that HCV related deaths in the canton of Bern could increase by 50% in 2030. The same trend was projected for the incidence of HCV-related HCC as well as for decompensated cirrhosis. Similar challenges have been described by Quanadli and Rotzinger [28] in a perspective article illustrating three examples of non-COVID-19 patients with advanced stage of infectious diseases who were admitted several weeks later than they would have been in usual practice. The authors described these cases as “conditions [that] have not been seen in western countries for decades”. The lack of timely management of HCV could have fatal consequences for some patients in the coming years.

While the future treatment paradigm in this “worst case” scenario is hypothetical, it was important to model both aspirational and conservative treatment paradigms to evaluate the range of possible disease outcomes. Special efforts should be taken to avoid delays in screening, linkage to care and treatment, even during a global pandemic and even if a duplication of these efforts is required to reach the elimination targets. In a recent analysis from Blach *et al*. [29], the authors also conclude that the impact of COVID-19 will extend beyond the direct morbidity and mortality and that policy makers should prioritize HCV programs as soon as possible.

### Limitations

There are several limitations to this analysis that may affect the outcomes. Despite efforts to find a consensus, the most important limitation remains the uncertainty regarding the true prevalence and incidence of HCV (and therefore also the uncertainty in the diagnosis rate). To our knowledge, no recent prevalence studies have been conducted in the general Swiss population. Large-scale screening could be done to confirm the prevalence number, while also increasing the number of patients diagnosed with HCV. Another limitation is that we assume that the effort in place to reduce new infections, e.g., harm reduction strategies as well as access to care and treatment, will continue in the future years. We also assume that the number of patients diagnosed and treated each year will remain the same into the future under the COVID and 2020 Base scenarios. This may be optimistic as efforts to diagnose will become increasingly challenging as the pool of infections is depleted through treatment. Moreover, no treatment data are registered in Switzerland, hence the exact numbers of treated and cured patients in the canton of Bern are unknown as well. The final limitation is linked to the model itself; the progression rates by age, sex and fibrosis score were retrospectively calculated using historical data.

## Conclusion

This analysis shows that the elimination of HCV in the Swiss canton of Bern before 2030 is still possible with a local and differentiated approach using a cantonal micro-elimination plan; however, not at the current pace of screening, linkage to care and treatment. The potential COVID-19 impact on treatment is substantial but can be overcome. In order to reach the “Swiss Hepatitis Strategy” goal there should be a greater focus on more rapidly increasing screening and improving linkage to care and treatment of hepatitis C.

## Supporting information

S1 File (XLSX)

S1 Appendix HCV disease burden model, forecasting viremic prevalence. (Adapted from supplements of [1] and [2]).
(DOCX)

S2 Appendix The Delphi process. (Adapted from supplement of [1]).
(DOCX)

S3 Appendix Sensitivity and uncertainty analyses. Key drivers of uncertainty in the 2019 prevalence.
(DOCX)
