# Supplementary material for: Modelling the microelimination of chronic hepatitis C in the canton of Bern, Switzerland: Reaching the Swiss Hepatitis Strategy goals despite the impact of the COVID 19 pandemic
Source: PLoS One. 2022 Aug 12;17(8):e0272518. doi: 10.1371/journal.pone.0272518 (PMC9374235; doi:10.1371/journal.pone.0272518)
Supplement: S2 Appendix — (Adapted from supplement of [1]). (DOCX) [file pone.0272518.s003.docx]

**Appendix 2: The Delphi Process**(Adapted from supplement of [1])

**Phase 1: Data Gathering**

Identify country experts who are willing to collaborate

- Experts were identified through HCV-related scientific contributions, or through referrals and personal acquaintance. Panels consisted of hepatologists, infectiologists and an addiction medicine specialist.
- For the Delphi meeting, we contacted the cantonal centers with high interest to better understand the situation in the canton.
- We invited 4 centers to participate, 3 of which responded positively (the remaining one declined due to lack of time related to the COVID-19 pandemic).
- We organized two Delphi meetings (September 2, 2020 and November 11,2020) with 3 and 2 cantonal experts, respectively.
- Origin of the HCV experts:
  - University: Inselspital Bern (hepatologist)
  - Hospital in Thun (gastroenterologist)
  - KODA, heroin assisted treatment center in Bern and university hospital Inselspital Bern (infectiologist)
- Final data were analyzed, and outputs were reviewed by the president of the Swiss Hepatitis association, Internal medicine specialist, medical director of the ARUD center for addiction medicine, Zürich (not part of the Delphi meetings)

Literature Search (August 2020)

- Reviewed the internal database for previously identified sources
- Reviewed online sources (FOPH & WHO) to capture non-indexed sources using the keyword: Hepatitis C OR HCV
  - Review of the FOPH website (<https://www.bag.admin.ch/bag/de/home.html>)
  - Review of the WHO website (<https://www.who.int/>)
- Ran a literature search from 2013 forward to identify recent publications
  - PubMed/MEDLINE search was done in August 2020:
    - (HCV) AND (Hepatitis C) AND (Switzerland) AND (prevalence) AND (("2013"[Date - Publication] : "3000"[Date - Publication]))
    - (HCV) AND (Hepatitis C) AND (Switzerland) AND (prevalence) AND (Bern) AND (("2013"[Date - Publication] : "3000"[Date - Publication]))
  - Selection of relevant and actual literature with title and abstract
- Summarized input data available through the literature
- Filled in the draft model based on published data or extrapolate inputs from countries with data when data are missing (as a placeholder)
- Scheduled meetings with experts (Phase 2)

**Phase 2: Country Meetings and Modeling**

Expert Meeting 1 (2.5 hours)

Objectives:

- Provide a background on the project, model, and methodology
- Review data identified in literature search and highlight gaps in data
- Request data in local non-indexed journals, unpublished data, and any other available data (e.g., hospital-level data) that can be used to fill the gaps
- Gain agreement on countries that can be used for extrapolation when no local data are available

Follow-up with Experts Post Meeting 1

- Sent the list of remaining action items to experts
- Followed up with experts to collect missing data, unpublished data, and raw hospital or registry-level data
- Analyzed data and sent to experts for approval

Disease Burden Modeling

- Populated disease burden model with inputs and calibrated model to empirical data
- Developed and ran scenarios requested by experts
- Scheduled second meeting
- Developed a slide deck summarizing all inputs

Expert Meeting 2 (2.5 hours)

Objectives

- Review and gain agreement on all inputs as well as data provided by experts since meeting 1
- Review results of analyses of any new data provided
- Present scenarios discussed with experts and review results and insights
- Agree on final strategies that would be considered

**Phase 3: Follow-up Analyses**

Follow-up Analyses

- Updated model as necessary and sent results to experts
- Provided support to address follow-up questions
- Updated analysis as new information became available
- Locked down inputs and outputs as approved

**Reference**

1. Polaris Observatory HCV Collaborators. Global prevalence and genotype distribution of hepatitis C virus infection in 2015: a modelling study. Lancet Gastroenterol Hepatol. 2017;2(3):161-76. Epub 2017/04/14. doi: 10.1016/s2468-1253(16)30181-9. PubMed PMID: 28404132.
