# Supplementary material for: Modelling the microelimination of chronic hepatitis C in the canton of Bern, Switzerland: Reaching the Swiss Hepatitis Strategy goals despite the impact of the COVID 19 pandemic
Source: PLoS One. 2022 Aug 12;17(8):e0272518. doi: 10.1371/journal.pone.0272518 (PMC9374235; doi:10.1371/journal.pone.0272518)
Supplement: S3 Appendix — Key drivers of uncertainty in the 2019 prevalence. (DOCX) [file pone.0272518.s004.docx]

**Appendix 3: Sensitivity and uncertainty analyses**

Key drivers of uncertainty in the 2019 prevalence

*The low and high listed on this chart indicate the low and high values used in the sensitivity analysis around the input value*
